# Prognostic Model of Eleven Genes Based on the Immune Microenvironment in Patients With Thymoma

**DOI:** 10.3389/fgene.2022.668696

**Published:** 2022-02-11

**Authors:** Ying Yang, Liqing Xie, Chen Li, Liangle Liu, Xiuzhi Ye, Jianbang Han

**Affiliations:** ^1^ Stroke Center and Departement of Neurology, The First Affiliated Hospital, Jinan University, Guangdong, China; ^2^ The Third Affiliated Hospital of Wenzhou Medical University, Wenzhou, China

**Keywords:** thymoma, immune microenvironment, prognostic model, genes, biomarker

## Abstract

**Purpose:** The pathogenesis of thymoma (THYM) remains unclear, and there is no uniform measurement standard for the complexity of THYM derived from different thymic epithelial cells. Consequently, it is necessary to develop novel biomarkers of prognosis estimation for patients with THYM.

**Methods:** Consensus clustering and single-sample gene-set enrichment analysis were used to divide THYM samples into different immunotypes. Differentially expressed genes (DEGs) between those immunotypes were used to do the Kyoto Encyclopedia of Genes and Genomes analysis, Gene Ontology annotations, and protein-protein interaction network. Furthermore, the survival-related DEGs were used to construct prognostic model with lasso regression. The model was verified by survival analysis, receiver operating characteristic curve, and principal component analysis. Furthermore, the correlation coefficients of stemness index and riskscore, tumor mutation burden (TMB) and riskscore, drug sensitivity and gene expression were calculated with Spearman method.

**Results:** THYM samples were divided into immunotype A and immunotype B. A total of 707 DEGs were enriched in various cancer-related or immune-related pathways. An 11-genes signature prognostic model (*CELF5, ODZ1, CD1C, DRP2, PTCRA, TSHR, HKDC1, KCTD19, RFX8, UGT3A2,* and *PRKCG*) was constructed from 177 survival-related DEGs. The prognostic model was significantly related to overall survival, clinical features, immune cells, TMB, and stemness index. The expression of some genes were significantly related to drug sensitivity.

**Conclusion:** For the first time, a prognostic model of 11 genes was identified based on the immune microenvironment in patients with THYM, which may be helpful for diagnosis and prediction. The associated factors (immune microenvironment, mutation status, and stemness) may be useful for exploring the mechanisms of THYM.

## Introduction

Thymoma (THYM), which can be benign or malignant, is one of the most common anterior mediastinal tumors (accounting for approximately 20%) and has unique clinicopathological characteristics. One-third of patients with THYM have oppression symptoms such as chest pain, superior vena cava syndrome, cough, or dysphagia (Weissferdt et al., 2018). THYM is closely associated with autoimmunity (THYM-associated multiorgan autoimmunity), of which myasthenia gravis (30–45% patients with THYM) is the most common autoimmune disorder (Yamamoto et al., 2020). The pathological type and Masaoka stage affect the prognosis of patients with THYM, which are complex. The classification of THYM by the World Health Organization (WHO) is largely determined by the proportion of lymphocytes, including type A (oval or fusiform shape with a lower lymphocyte count), type B (epithelioid shape; type B1, B2, and B3), and type AB (combination of different cell types). Among these pathological types, type B3 is considered more invasive than the others (Ishihara et al., 2020). The Masaoka stage classification of THYM, including stages I, IIA, IIB, III, IVA, and IVB, is largely determined by infiltration of the thymic tumor envelope and metastasis (generally to the pleura, liver, bones, or brain). The prognosis of stage III or IV THYM is considered to be much worse than that of other stages (Yang et al., 2020). Surgery is still the main method of THYM therapy, but preoperative or postoperative chemotherapy and radiotherapy may be necessary for tumors that are apparently invasive and large (Kim et al., 2020). Although survival rates for patients with THYM are consistently higher than those for other types of cancer, the complexity of THYM types results in a lack of uniform measurement standards. Currently, the treatment of THYM comprising different tissue types still lacks effective guidelines in some cases, owing to histological diversity (Li et al., 2019).

Currently, immunotherapy has been widely used in the treatment of advanced-stage tumors and has shown efficacy in a variety of aggressive tumor species. Inhibitors of programmed death 1 (PD-1)/programmed death ligand 1 (PD-L1), such as pembrolizumab and nivolumum, help to decrease the immune escape of tumor cells (Kalbasi and Ribas, 2020). One study reported the application of PD-L1 antibody (Avelumab) treatment in advanced-stage THYM. The results showed that in patients with THYM, the population of immune cell subsets was different between responders and non-responders. Responders had lower proportions of regulatory dendritic cells, T cells, B cells, and natural killer cells prior to the treatment (Rajan et al., 2019). The thymus, an immune organ, plays an important role in inducing the development, differentiation, and maturation of T-lymphocytes (Zdrojewicz et al., 2016). The association between THYM and paraneoplastic autoimmunity is clear. Various autoimmune diseases are common complications of THYM, including myasthenia gravis, thyroid disorders, Isaac’s syndrome, erythroblastopenia, limbic encephalitis, systemic lupus erythematosus, inflammatory myopathies, autoimmune hepatitis, Good’s syndrome, Morvan’s syndrome, and bullous skin diseases (Hung et al., 2019). The relationship among the expression of tumor-associated genes, related pathways, and immune cells based on different immunotypes may provide novel insights into immunotherapy of THYM (Rappaport et al., 2020). With the development of the immune system–mediated killing of tumor cells, research has begun to show that fibrosis, gene expression, angiogenesis, and immune cells in the tumor microenvironment are dysregulated and influenced in THYM, and some of these factors have been demonstrated to be associated with the degree of malignancy and clinical metastasis (Sato et al., 2020). For example, type B2/B3 THYM showed a high possibility of unfavorable prognosis, and the heterogeneity of PD-L1 expression was significantly different between type A/AB/B1 and type B2/B3 (Chen et al., 2020). Further studies investigating the alteration of immune subtypes would be meaningful in developing immunotherapy for THYM.

**FIGURE 1:**
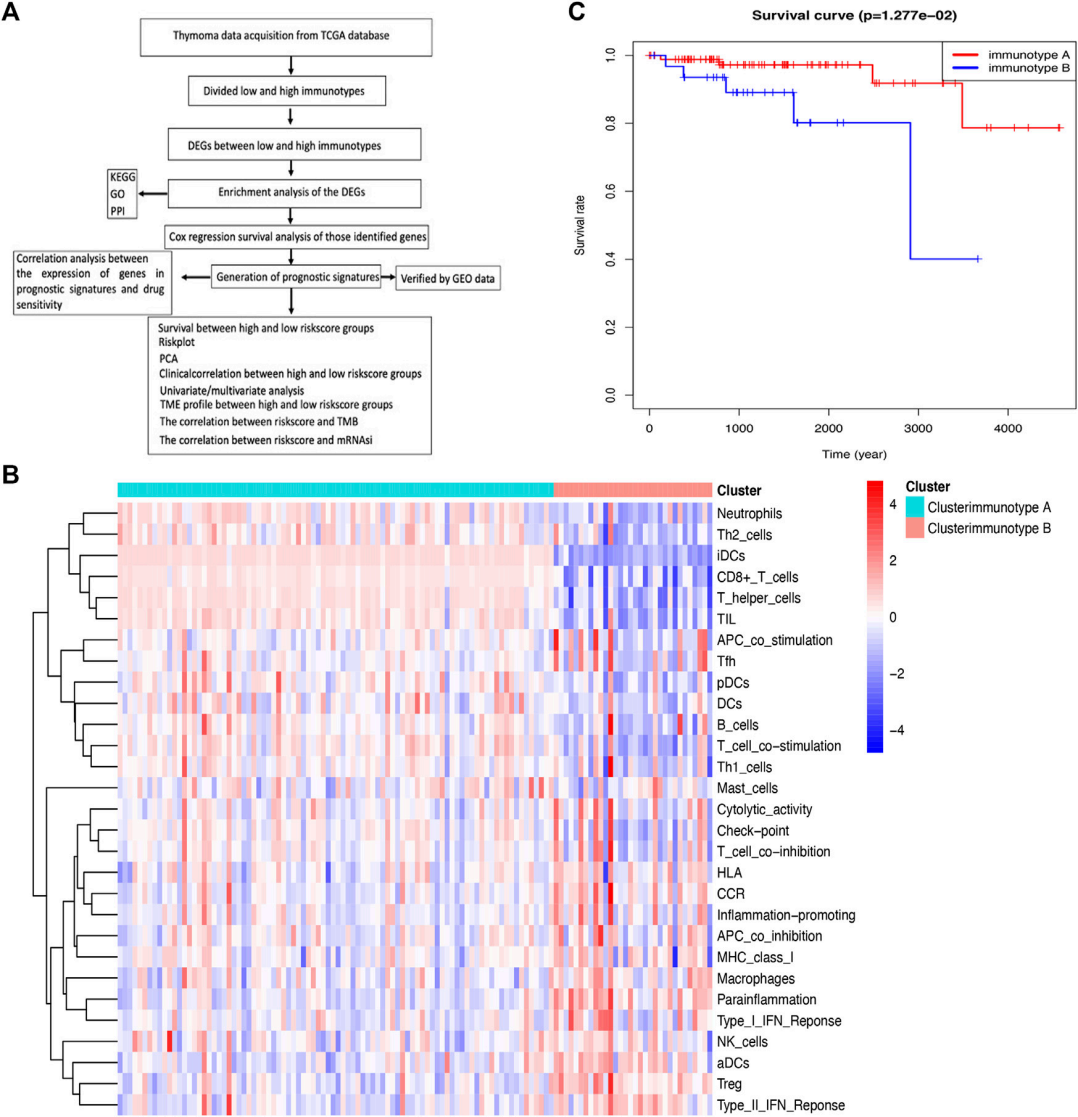
Comparison of immune cell infiltration between immunotypes A and B in THYM. **(A)** Flow chart for the identification of immune signatures in THYM. **(B)** The heatmap shows that the THYM samples were classified as immunotype A or B according to immune cell infiltration. **(C)** Kaplan–Meier curves show that the immunotype A had greater mortality than the immunotype B.

In this study, immune status stratification was used to divide cases into different immunotypes, and differentially expressed genes (DEGs), enriched pathways, and related biological functions were explored, which suggested some potential mechanisms of immune-related events. A multiple gene model based on the immune microenvironment was constructed to predict the prognosis of patients with THYM. The calculated risk score, which combines the corresponding clinical characteristics, will be beneficial in clinical applications of the prognostic model. The comparison of immune cells, stemness indices, tumor mutation burden (TMB), and drug sensitivity between high- and low-risk score groups provides insight into several aspects of relationships in the field of immunological studies. Nevertheless, a gene signature for the prognosis could help to elucidate the pathogenesis of THYM in the future. The flow chart is shown in Figure 1A.

## Materials and Methods

### RNA-Seq Data and Clinical Data in THYM

RNA-seq data and clinical data in THYM were downloaded from the TCGA database (https://portal.gdc.cancer.gov/), including the expression of 20,530 mRNA and eight clinical features, including age at initial pathologic diagnosis (from 17 to 84 years old), sex (female and male), histological type (Type A, Type AB, Type B1, Type B2, Type B3, and Type C), history of myasthenia gravis (yes or no), Masaoka stage (I, IIa, IIb, III, and IV), cancer status (with tumor or tumor free), new tumor event after initial treatment (yes or no), and radiation therapy (yes or no), in 121 samples with THYM. The epigenetically regulated mRNA expression–based stemness index (EREG-mRNAsi) was confirmed to be related to the biological processes of stemness and tumor state. Cell stemness was evaluated using the EREG-mRNAsi, which was obtained from the UCSC Xena datasets (https://xenabrowser.net/datapages/). The EREG-mRNAsi ranged from 0 to 1, of which values closer to 1 represented stronger stemness characteristics. The TMB is the number of mutations that cause amino acid changes in the coding region per million bases in the tested DNA. The TMB is a new important biomarker owing to its predictive value of the efficacy of cancer immunotherapy. The Maftools R package (https://www.bioconductor.org/packages/release/bioc/html/maftools.html) was used to calculate TMB according to somatic mutation data, which were downloaded from the UCSC Xena datasets (https://xenabrowser.net/datapages/).

### Division of Samples into Different Immunotypes Based on Immune Cell Infiltration

A single-sample gene set enrichment analysis (ssGSEA) was used to evaluate the proportion of immune cell infiltration in THYM via the GSEABase R package (https://www.bioconductor.org/packages/release/bioc/html/GSEABase.html). In total, 29 types of immune factors in the tumor microenvironment were summarized, including aDCs, APC co-inhibition, APC co-stimulation, B cells, CCR, CD8^+^ T cells, checkpoint, cytolytic activity, DCs, HLA, iDCs, inflammatory cells, macrophages, mast cells, MHC class I, neutrophils, NK cells, para-inflammation, pDCs, T-cell co-inhibition, T-cell co-stimulation, T helper cells, Tfh, Th1 cells, Th2 cells, TIL, Treg, Type I IFN response, and Type II IFN response. Samples with THYM were divided into different immunotypes (A and B) based on immune cell infiltration via the ConsensusClusterPlus R package (https://bioconductor.org/packages/release/bioc/html/ConsensusClusterPlus.html). The Kaplan–Meier (KM) survival analysis curve was plotted to show the difference in overall survival rate between immunotype A and immunotype B (*p* < .05 indicated a significant difference).

### Identification of DEGs Between Immunotype A and Immunotype B in THYM

The DEGs between immunotypes A and B [*p* < .05, false discovery rate (FDR) ≤ .05, and log (fold change) ≥ 1] in THYM were analyzed with the limma package (http://web.mit.edu/∼r/current/arch/i386_linux26/lib/R/library/limma/html/01Introduction.html). The immune-related genes (IRGs) were downloaded from the Immunology Database and Analysis Portal (IMMPORT) website (https://immport.niaid.nih.gov/home), and a Venn diagram was used to extract overlapping DEGs between immunotypes A and B and IRGs (https://bioinfogp.cnb.csic.es/tools/venny/index2.0.2.html).

### Gene Ontology, Signaling Pathways, and Protein-Protein Interaction Network of DEGs in THYM

Analysis of Gene Ontology (GO) terms was conducted in R (https://ucdavis-bioinformatics-training.github.io/2018-June-RNA-Seq-Workshop/friday/enrichment.html) to identify DEGs between immunotype A and immunotype B, including biological process (BP), molecular function (MF), and cellular component (CC). BP refers to an orderly biological process with multiple steps, such as cell growth, differentiation, and maintenance; apoptosis; and signal transduction. MF typically refers to the function of a single gene product, such as binding activity or catalytic activity. CC describes the position of gene products in the cell, such as the endoplasmic reticulum, nucleus, or proteasome. The GO enrichment *p* value was calculated (*p* < .05), and the FDR value was adjusted using the Benjamini–Hochberg method for multiple testing (adjusted *p* < .05). The Kyoto Encyclopedia of Genes and Genomes (KEGG) pathways were analyzed using the DAVID website (https://david.ncifcrf.gov/home.jsp), and pathways with *p* < .05 were significant. The protein-protein interaction (PPI) network of the DEGs in THYM was constructed using the String database (https://string-db.org/). The PPI network was analyzed with Cytoscape software to obtain hub molecules via the cytoHubba app (*p* < .05).

### Construction and Verification of Lasso Regression in THYM

The overall survival (OS)-related DEGs of THYM were analyzed with the Cox regression survival package in R (http://rstudio-pubs-static.s3.amazonaws.com/5896_8f0fed2ccbbd42489276e554a05af87e.html). A hazard ratio (HR) >1 indicates that the research gene is a risk factor, whereas HR <1 indicates that it is a protective factor. The OS-related DEGs were further used to construct a least absolute shrinkage and selection operator (lasso) regression using the glmnet package in R (https://www.geeksforgeeks.org/lasso-regression-in-r-programming/). A multiple-gene signature prognostic model was constructed from survival-related DEGs, and the risk score for each sample was calculated according to the prognostic model. All THYM cases could be divided into high- and low-risk score groups based on the median value of the risk score. The KM survival curve was used to compare the relevance of OS between risk score groups (*p* < .05). The distribution of survival status (dead or alive) between different risk score groups was further used to describe the validity and discernment capability of OS. The multiple receiver operating characteristic (ROC) curve was used to test the sensitivity and specificity of the prognostic model in R (https://cran.r-project.org/web/packages/ROCR/index.html). The principal component analysis (PCA) package in R (https://www.datacamp.com/community/tutorials/pca-analysis-r) was used to evaluate whether the constructed prognostic model could distinguish the two different groups of THYM. The verification data were downloaded from the GEO database (https://www.ncbi.nlm.nih.gov/geo/query/acc.cgi?acc=GSE29695). RNA was extracted from fresh frozen tumors of 36 patients with THYM, and follow-up data were available (GSE29695). The corresponding gene expression and clinical data of the prognostic model were selected from GSE29695, and all cases were divided into different risk-score groups according to the prognostic model constructed from the TCGA database.

### Correlation of Risk Score With Clinical Characteristics, Immune Cells, TMB, EREG-mRNAsi, and Drug Sensitivity

The clinical characteristics included age at initial pathologic diagnosis (17–84 years), sex (female or male), histological type (Type A, Type B1, Type B2, Type B3, Type AB, and Type C), history of myasthenia gravis (yes or no), Masaoka stage (stage I, stage IIa, stage IIb, stage III, and stage IV), cancer status (with or without tumor), new tumor event after initial treatment (yes or no), and radiation therapy (yes or no). The statistically significant differences in clinical characteristics between the high- and low-risk score groups were plotted using the pheatmap package in R (https://rdrr.io/bioc/TRONCO/man/pheatmap.html). Univariate and multivariate Cox regression survival models were also used to evaluate risk factors of THYM, including some clinical characteristics and risk scores (https://www.bioconductor.org/packages/release/bioc/vignettes/RTNsurvival/inst/doc/RTNsurvival.html). The proportions of immune cells in THYM were calculated and downloaded from CIBERSORT (https://cibersortx.stanford.edu) with the algorithm running 1,000 permutations based on the mRNA expression profiles of THYM, including 22 human immune cell phenotypes (naïve CD4 T cells, CD8 T cells, gamma T cells, resting memory CD4 T cells, naïve B cells, memory B cells, activated memory CD4 T cells, delta, regulatory T cells (Tregs), plasma cells, follicular helper T cells, M1 macrophages, M2 macrophages, resting NK cells, activated NK cells, neutrophils, M0 macrophages, resting mast cells, resting dendritic cells, activated mast cells, activated dendritic cells, eosinophils, and monocytes). The significant difference in immune cells between the high- and low-risk score groups was tested with an independent sample *t*-test (*p* < .05). The identified immune cells between the two groups were further analyzed via correlation analysis (https://www.r-graph-gallery.com/199-correlation-matrix-with-ggally.html). Correlation analyses between the risk score and TMB and between the risk score and EREG-mRNAsi were performed using the Spearman method (*p* < .05). The drug sensitivity data of gene expression in the prognostic model were downloaded from CellMiner (https://discover.nci.nih.gov/cellminer/) and analyzed via the Spearman method (*p* < .05) using the Corrplot package in R (https://www.rdocumentation.org/packages/corrplot/versions/0.84).

### Sample Collection and Immunohistochemistry

An independent set of formalin-fixed paraffin-embedded tissue specimens, namely, 3 cases of NC, and 3 cases of THYM, were obtained from the Department of Pathology of the first affiliated hospital, at Jinan University and used for immunohistochemical analysis. Immunohistochemistry was performed according to the procedure. Briefly, 4-μm-thick tissue sections were deparaffinized, rehydrated, and treated with an antigen retrieval solution (10 mmol/L sodium citrate buffer, pH 6.0). The sections were incubated with anti-CD1C (1:250; Abcam) overnight at 4°C and then incubated with biotinylated secondary antibody followed by addition of avidin-biotin peroxidase. Finally, tissue sections were incubated with 3′,3′-diaminobenzidine until a brown color developed, and they were counterstained with Harris’ modified Hematoxylin. In negative controls primary antibodies were omitted.

## Results

### Immunotypes were Significantly Associated With THYM

Along with the development of tumor immunology and molecular biotechnology, immunotherapy has become crucial in the combined therapy of tumors. The flow chart is summarized in Figure 1A. In this study, the overall bioinformatics analysis revealed the association among cancer stemness, drug sensitivity, gene mutations, and immune status in THYM. All THYM samples were divided into different immunotypes (immunotype A = 87 cases; immunotype B = 33 cases) based on various inflammatory environmental factors (aDCs, APC co-inhibition, APC co-stimulation, B cells, CCR, CD8^+^ T cells, checkpoint, cytolytic activity, DCs, HLA, iDCs, inflammatory promoting, macrophages, mast cells, MHC class I, neutrophils, NK cells, para-inflammation, pDCs, T-cell co-inhibition, T-cell co-stimulation, T helper cells, Tfh, Th1 cells, Th2 cells, TIL, Treg, Type I IFN response, and Type II IFN response) from ssGSEA (Supplementary Table S1 and Supplementary Table S2). The cluster heatmap indicated that the immunotype A cluster contained a higher proportion of immune factors such as neutrophils, Th2 cells, TIL, iDCs, CD8^+^ T cells, and TIL than did the immunotype B cluster. The immunotype B cluster contained a higher proportion of immune factors such as macrophages, para-inflammation, NK cells, Treg, Type II IFN response, and aDCs than did the immunotype A cluster (Figure 1B). Moreover, the survival analysis indicated that the immunotype A cluster had a significantly better survival prognosis than did the immunotype B cluster (*p* = 1.277e-02 in Figure 1C).

### DEGs Between Immunotype A and Immunotype B of THYM

**FIGURE 2:**
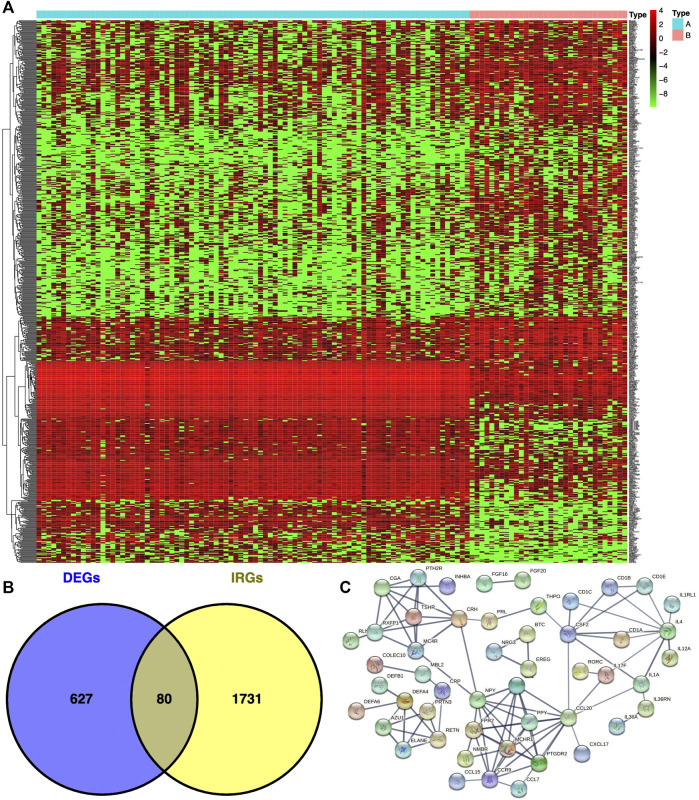
Differentially expressed genes (DEGs) and immune-related genes (IRGs) between immunotypes A and B in THYM. **(A)** The heatmap shows DEGs between immunotypes A and B in THYM. **(B)** The overlap between DEGs and IRGs in THYM. **(C)** Protein-protein interaction (PPI) network of differently expressed IRGs in THYM.

In total, 707 DEGs were identified between immunotype A and immunotype B of THYM using the limma package in R [*p* < .05, FDR ≤ .05, and log (fold change) ≥ 1], including 262 downregulated DEGs and 445 upregulated DEGs (Figure 2A). The top 10 downregulated DEGs included *OR10T2, HIST1H2BB, AOX2P, OR10K1, BHLHE23, C10orf129, HTR5A, NANOS2, PRL,* and *IL1F6*. The top 10 upregulated DEGs included *HOXA11, ADIG, PDC, CEACAM16, OR7C1, CNGB3, HOXD11, VIL1, MSLNL,* and *OR10K2* (Supplementary Table S3). The Venn diagram showed the overlapping genes between DEGs and IRGs, which included 80 genes (Figure 2B). The differentially expressed IRGs (DEIRGs) were classified into different categories, such as antigen processing and presentation, antimicrobials, chemokine receptors, chemokines, cytokine receptors, cytokines, natural killer cell cytotoxicity, and TCR signaling pathway (Supplementary Table S4). The DEIRGs showed significant PPI in the String network with a combined score greater than 0.7 (Figure 2C). Some DEIRGs in the PPI showed high combined scores and co-expression correlation scores, including *AZU1* and *PRTN3, ELANE* and *PRTN3, AZU1* and *DEFA4, AZU1* and *ELANE, CD1B* and *CD1E,* and *DEFA4* and *ELANE*.

### DEGs Between Immunotype A and Immunotype B Enriched in Various Functions and Pathways

**FIGURE 3:**
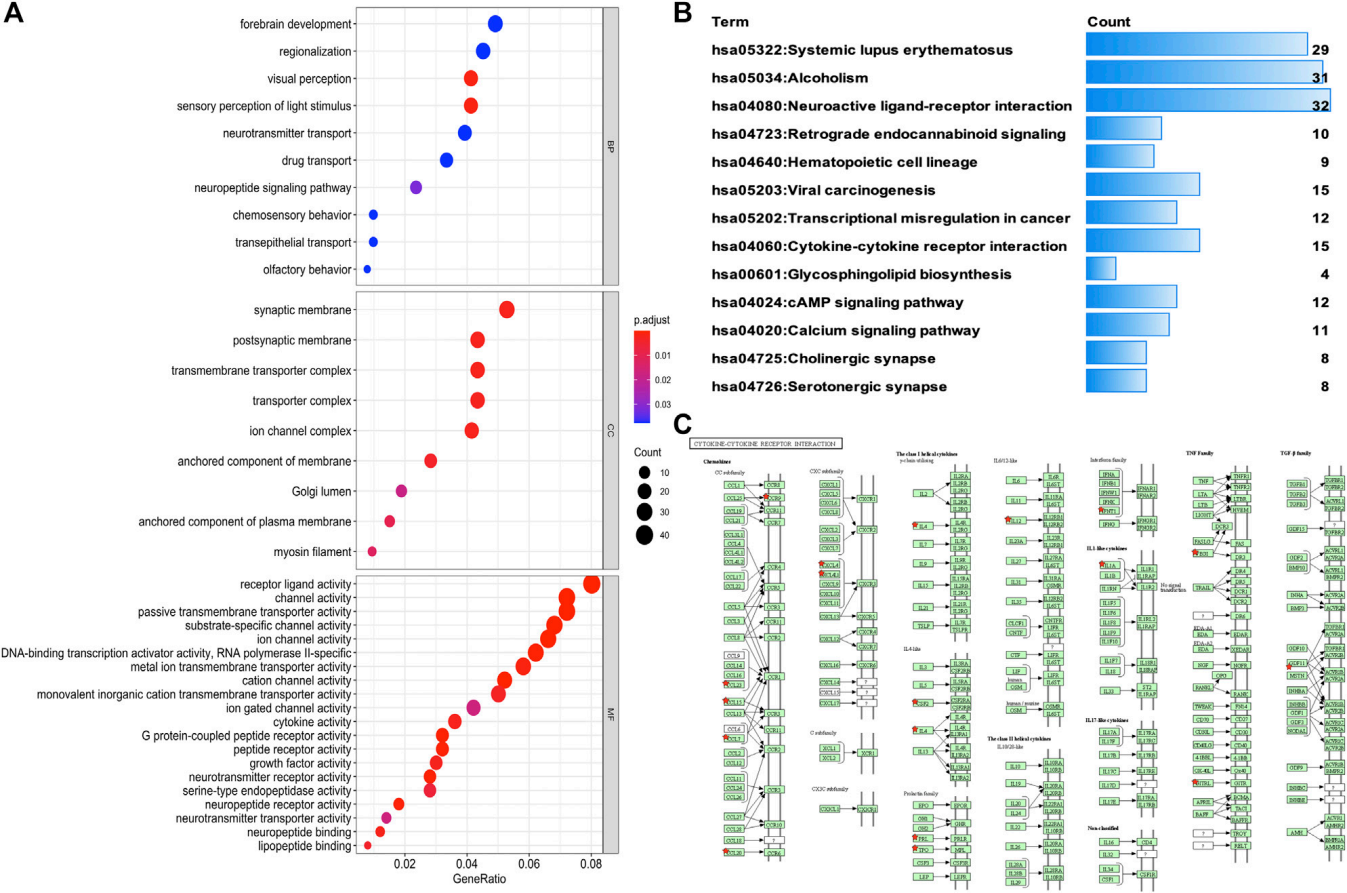
Functional characteristics and pathway enrichment analysis of DEGs between immunotypes A and B in THYM. **(A)** GO analysis (including biological process, cellular component, and molecular function) of DEGs between immunotypes A and B in THYM. **(B)** KEGG pathway enrichment analysis of DEGs between immunotypes A and B in THYM. **(C)** Cytokine-cytokine receptor interaction pathway enriched in THYM.

**FIGURE 4:**
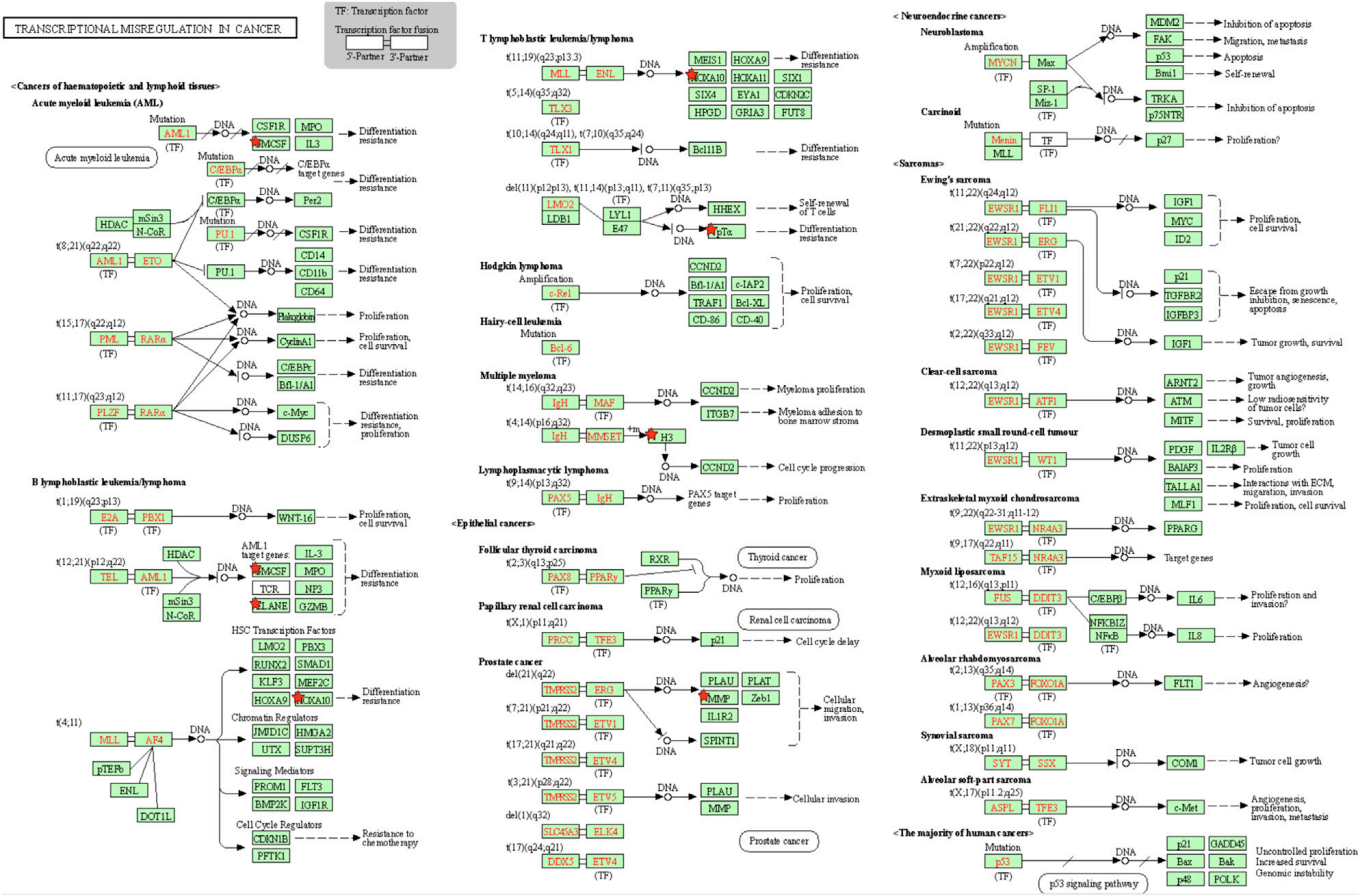
Transcriptional mis-regulation in cancer pathways enriched in THYM.

**FIGURE 5:**
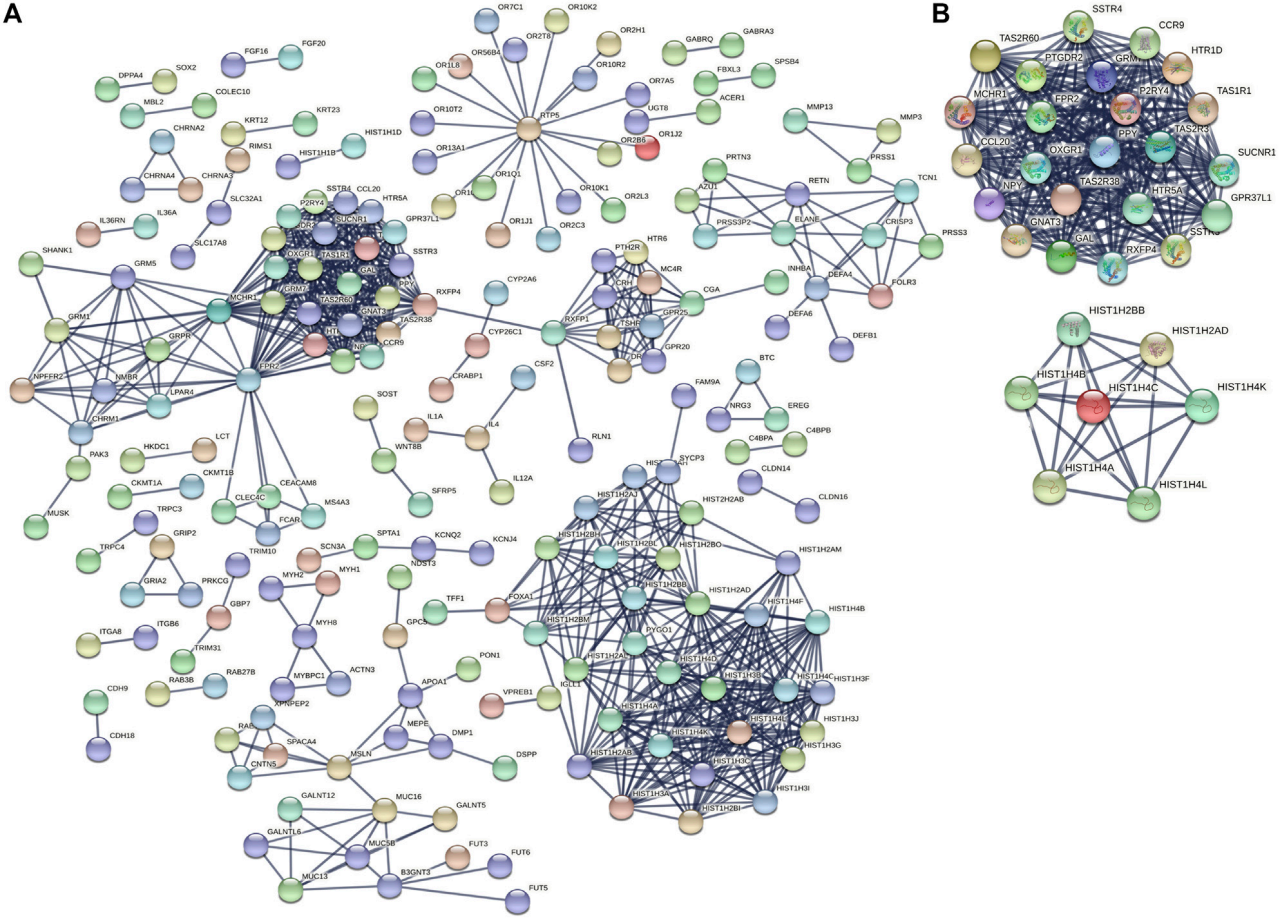
PPI networks and hub genes based on DEGs between immunotypes A and B in THYM. **(A)** PPI networks based on DEGs between immunotypes A and B in THYM. **(B)** Hub genes based on PPI network in THYM.

To identify the underlying biological characteristics that determine immune subtype, GO, KEGG, and PPI were used to identify DEGs between immunotype A and immunotype B in THYM. In total, 64 statistically significant GO enrichments (Supplementary Table S5) and 14 statistically significant KEGG enrichments (Supplementary Table S6) were found. The GO enrichment results indicated that the DEGs were closely related to visual perception, sensory perception of light stimulus, the neuropeptide signaling pathway, forebrain development, neurotransmitter transport, chemosensory behavior, drug transport, transepithelial transport, olfactory behavior, and regionalization according to BP. The DEGs mainly dominated cellular localization, including the synaptic membrane, postsynaptic membrane, ion channel complex, transmembrane transporter complex, transporter complex, anchored component of membrane, anchored component of plasma membrane, myosin filament, and Golgi lumen according to CC. The GO enrichment results indicated that the DEGs were closely related to receptor ligand activity, channel activity, cytokine activity, multiple peptidase activity, growth factor activity, and transmembrane transporter activity according to MF (Figure 3A). The GO analysis of DEGs provides insight into molecular localization and function in the initiation and progression of cancer. Those results could give some basic evidence for the further study. The KEGG enrichment results suggested that the DEGs were closely related to systemic lupus erythematosus, alcoholism, neuroactive ligand-receptor interaction, retrograde endocannabinoid signaling, hematopoietic cell lineage, viral carcinogenesis, transcriptional mis-regulation in cancer, cytokine-cytokine receptor interaction, glycosphingolipid biosynthesis, phototransduction, the cAMP signaling pathway, the calcium signaling pathway, cholinergic synapses, and serotonergic synapses (Figure 3B). Some immune processes and cancer-related pathways are of particular importance. For example, some crucial DEGs played important roles in the cytokine-cytokine receptor interaction pathway, including *TNFSF18, CSF2, TNFSF15, CCL20, INHBA, PRL, IL4, IL1A, CCL7, THPO, IFNE, CCR9, IL12A, PF4V1,* and *CCL15* (Figure 3C). Those enriched pathways and key molecules provide important information in THYM. Transcriptional mis-regulation in cancer pathways (*PTCRA, HOXA10, CSF2, HIST1H3A, HIST1H3J, MMP3, HIST1H3F, HIST1H3G, HIST1H3I, HIST1H3B, HIST1H3C,* and *ELANE*) was also found in THYM (Figure 4). These enriched signaling pathways revealed that some of the identified DEGs dominated hub locations in the pathway, which, to some extent, indicated potential mechanisms of THYM. The 12 mis-regulation genes were as key transcriptional molecules, and further verifications would obtain new mechanisms in THYM. The identified DEGs showed significant PPI in the String network with a combined score greater than 0.9 (Figure 5A and Supplementary Table S7). Some DEGs in the PPI showed high combined scores and co-expression correlation scores, such as *HIST1H2BO* and *HIST1H3B, HIST1H2BL* and *HIST1H2BO, HIST1H2BL* and *HIST1H2BM, HIST1H2BM* and *HIST1H2BO, DEFA4* and *ELANE, AZU1* and *ELANE, CKMT1A* and *CKMT1B, ELANE* and *PRTN3,* and *AZU1* and *PRTN3*. Moreover, the entire PPI network was analyzed using cytoHubba, and the hub module contained the top 30 hub genes including *TAS1R1, GAL, PTGDR2, GRM7, HIST1H2BB, HIST1H4K, HIST1H4L, SUCNR1, TAS2R3, TAS2R38, MCHR1, NPY, RXFP4, HIST1H4A, HIST1H4B, SSTR3, HIST1H4C, SSTR4, CCL20, GPR37L1, TAS2R60, GNAT3, HTR1D, P2RY4, HIST1H2AD, CCR9, OXGR1, HTR5A, PPY,* and *FPR2* (Figure 5B). The hub genes identified in the PPI network might play crucial roles in the regulation of the tumor process.

### Construction of a Prognostic Model and Verification With GEO Data for THYM

**FIGURE 6:**
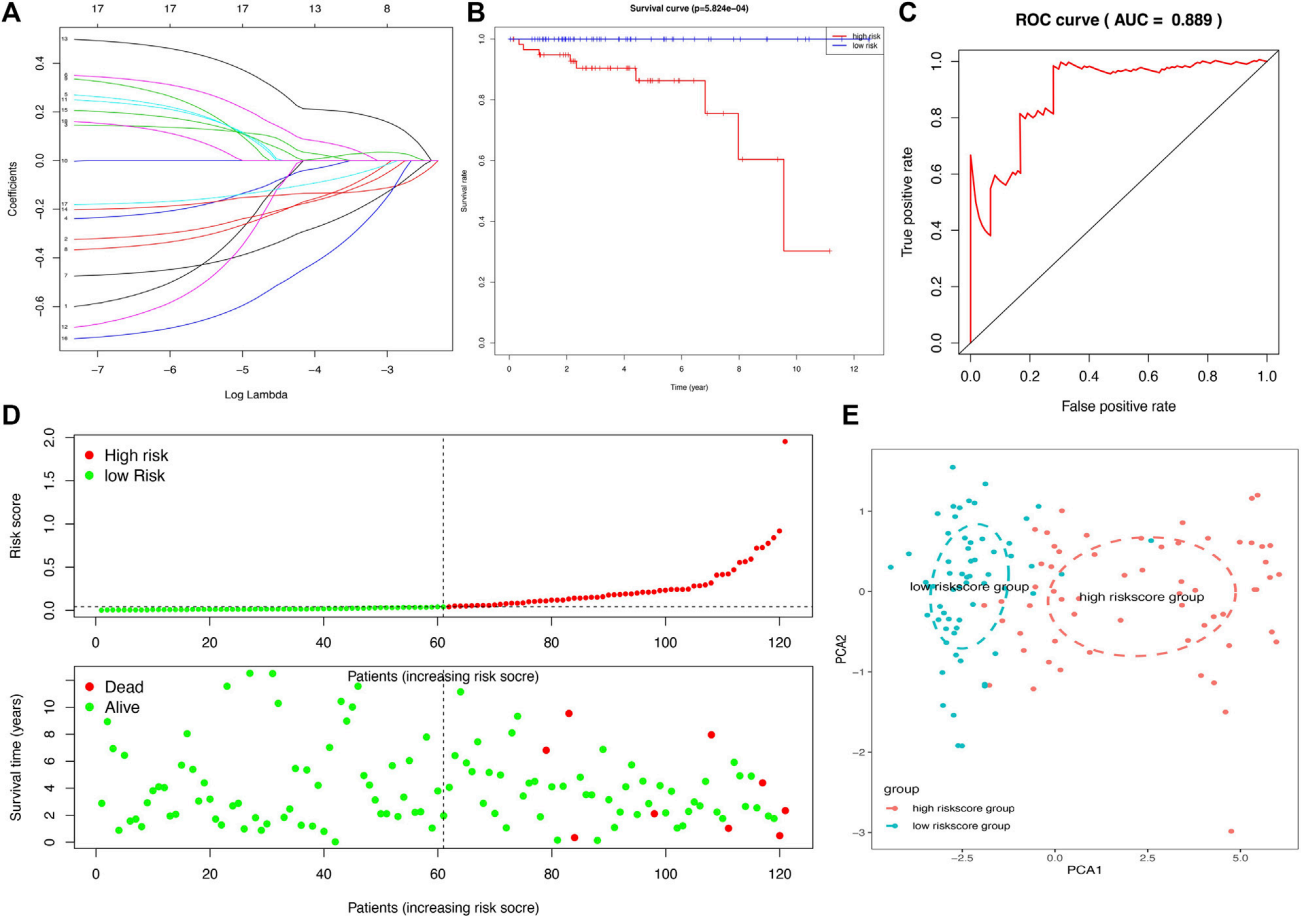
A lasso regression identified the prognostic model in THYM. **(A)** Lasso regression complexity was evaluated using lambda. **(B)** Overall survival analysis between high- and low-risk score groups from the 11-gene prognostic model in THYM. **(C)** ROC curve based on risk score in THYM. **(D)** The distribution of survival status (dead or alive) between high- and low-risk score groups in THYM. **(E)** PCA plot based on risk score in THYM.

In total, 11 survival-related DEGs, including *CELF5, ODZ1, CD1C, DRP2, PTCRA, TSHR, HKDC1, KCTD19, RFX8, UGT3A2,* and *PRKCG,* out of 177 OS-related DEGs were selected to construct an optimal lasso regression, in which log lambda ranged from −3 to −4 (Figure 6A and Supplementary Tables S8, S9). The intercept values of each identified genes in prognostic model, including CELF5 (−0.050), ODZ1 (0.002), CD1C (−0.036), DRP2 (−0.005), PTCRA (0.054), TSHR (0.066), HKDC1 (−0.014), KCTD19 (−0.010), RFX8 (−0.383), UGT3A2 (0.239), and PRKCG (−0.425). All samples of THYM were divided into high- and low-risk score groups according to the median value of the risk score (0.014). The survival rate was significantly different between the high- and low-risk score groups (*p* = 5.824e-04), which suggests that patients with a high risk score would have a poor prognosis (Figure 6B). Moreover, the distribution of survival status (dead or alive) was significantly different between the high- and low-risk score groups (Figure 6D). The area under the ROC curve (AUC), with a high value of 0.931, indicated that the prognostic model constructed by optimal lasso regression had high sensitivity and specificity (Figure 6C). Furthermore, the PCA plot compared satisfactory discrimination validity between the high- and low-risk score groups (Figure 6E). These analyses indicated that the prognostic model may be reliable in clinical application. GSE29695 data (Supplementary Table S10), including the clinical features of stage, relapse, and metastasis, were downloaded from the GEO database for exterior verification (Supplementary Table S11). The results showed that there were more patients with stage III and IV in the high-risk score group (*n* = 7) than in the low-risk score group (*n* = 4), and that there were more patients with metastasis in the high-risk score group (*n* = 6) than in the low-risk score group (*n* = 3).

**FIGURE 7:**
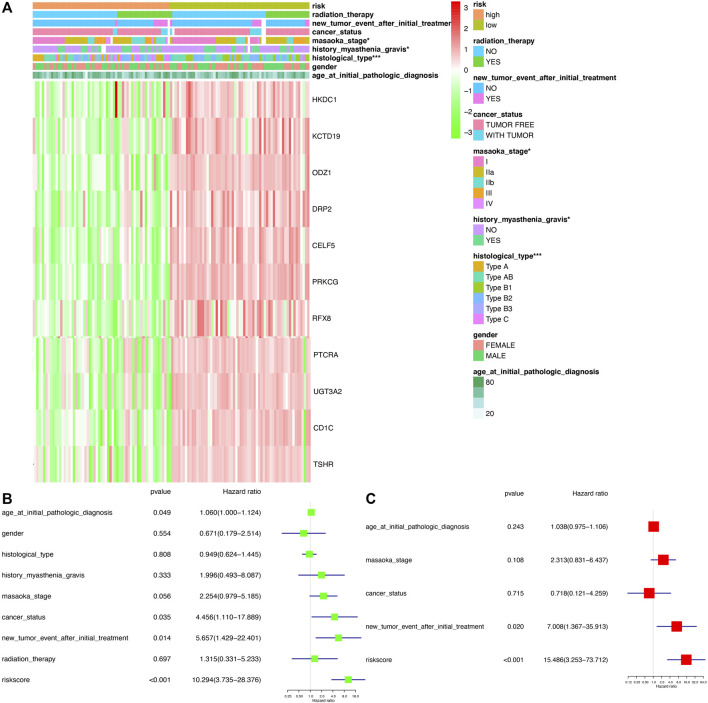
The clinical correlation between clinical features and risk score in THYM. **(A)** Heatmap of the clinical correlation between high- and low-risk score groups in THYM. **(B)** Univariate Cox regression analysis of risk factors in THYM. **(C)** Multivariate Cox regression analysis of risk factors in THYM. *p < .05, **p < .01, and ***p < .001.

The association between clinical data (age at initial pathologic diagnosis, gender, histological type, history of myasthenia gravis, Masaoka stage, cancer status, new tumor event after initial treatment, and radiation therapy) of THYM (Supplementary Table S12) and different risk-score groups was investigated with a heatmap. Some clinical features were significantly different between the two groups, including histological type, history of myasthenia gravis, and Masaoka stage (Figure 7A). A univariate Cox regression analysis revealed that age at initial pathologic diagnosis, cancer status, new tumor event after initial treatment, and risk score were significantly correlated with OS (Figure 7B). This indicated that the risk score of the prognostic model in THYM is one of the risk factors. Furthermore, a multivariate Cox regression analysis revealed that a new tumor event after initial treatment and risk score could be independent risk factors for THYM (Figure 7C). These results indicate that risk score might be a novel biomarker for evaluating the prognosis and survival status of THYM.

### Association of Risk Score With Immune Cells, TMB, Stemness, and Drug Sensitivity

**FIGURE 8:**
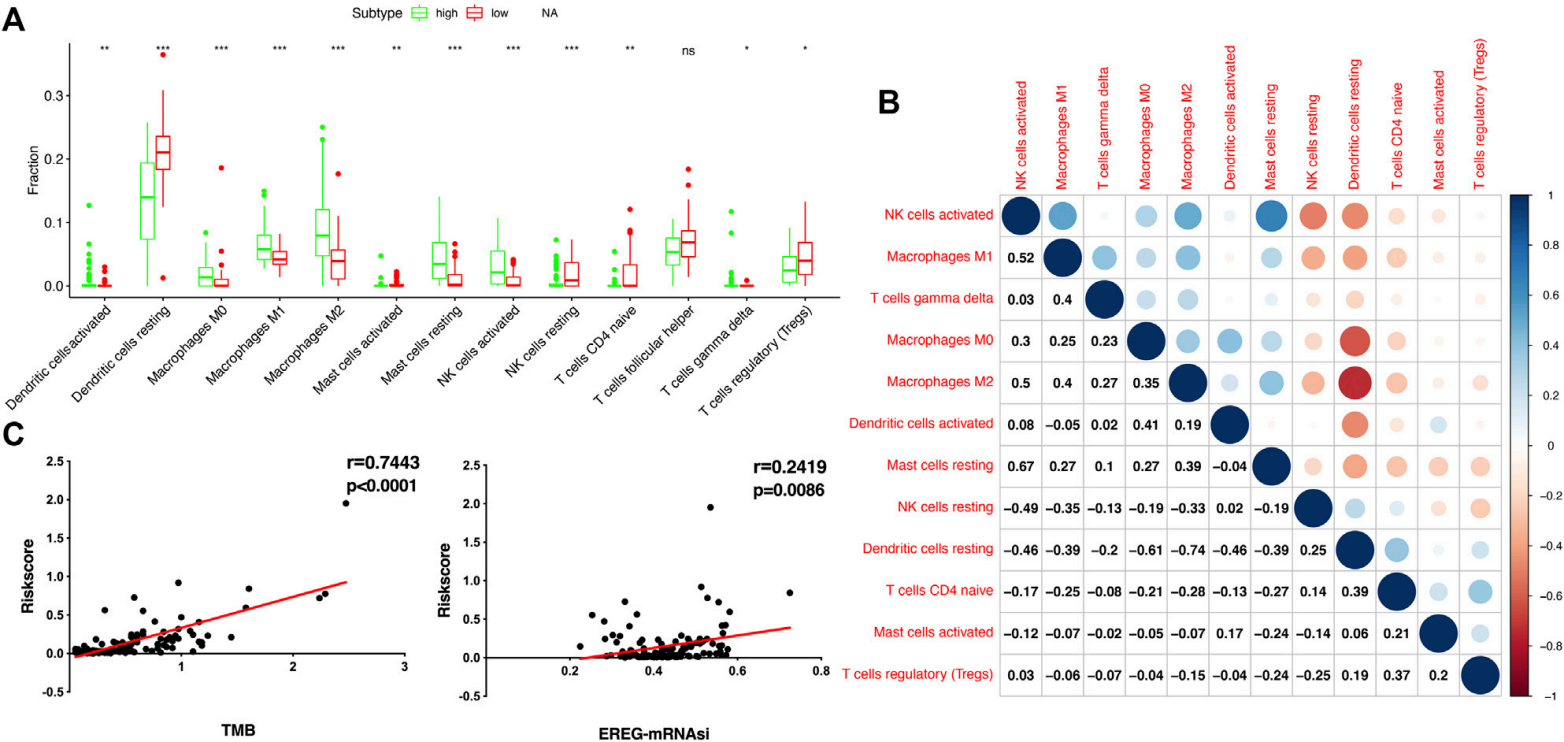
The association of immune cells, TMB, and stemness with risk score in THYM. **(A)** The boxplot shows the differences in 12 immune cells between high- and low-risk score groups in THYM. **(B)** The correlation of those 12 immune cells in THYM. **(C)** The association between TMB and risk score in THYM. **(D)** The association between stemness index and risk score in THYM.

**FIGURE 9:**
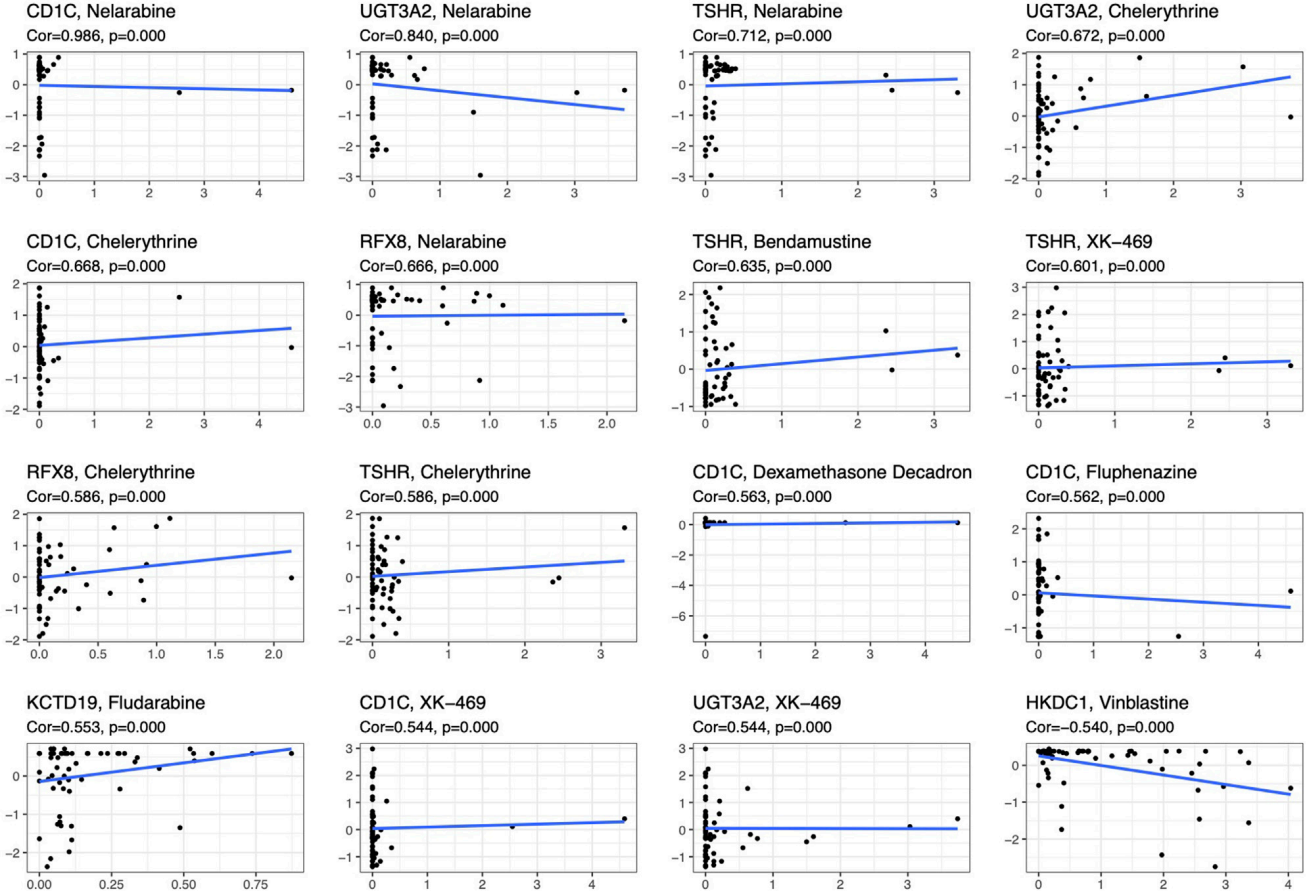
The significant correlation between gene expression in the prognostic model and sensitivity to drugs including Vinblastine, PX-316, Nelarabine, Bendamustine, Asparaginase, Cladribine, Chlorambucil, Rebimastat, Fludarabine, XK-469, Fluphenazine, Dexamethasone Decadron, and Chelerythrine.

**FIGURE 10:**
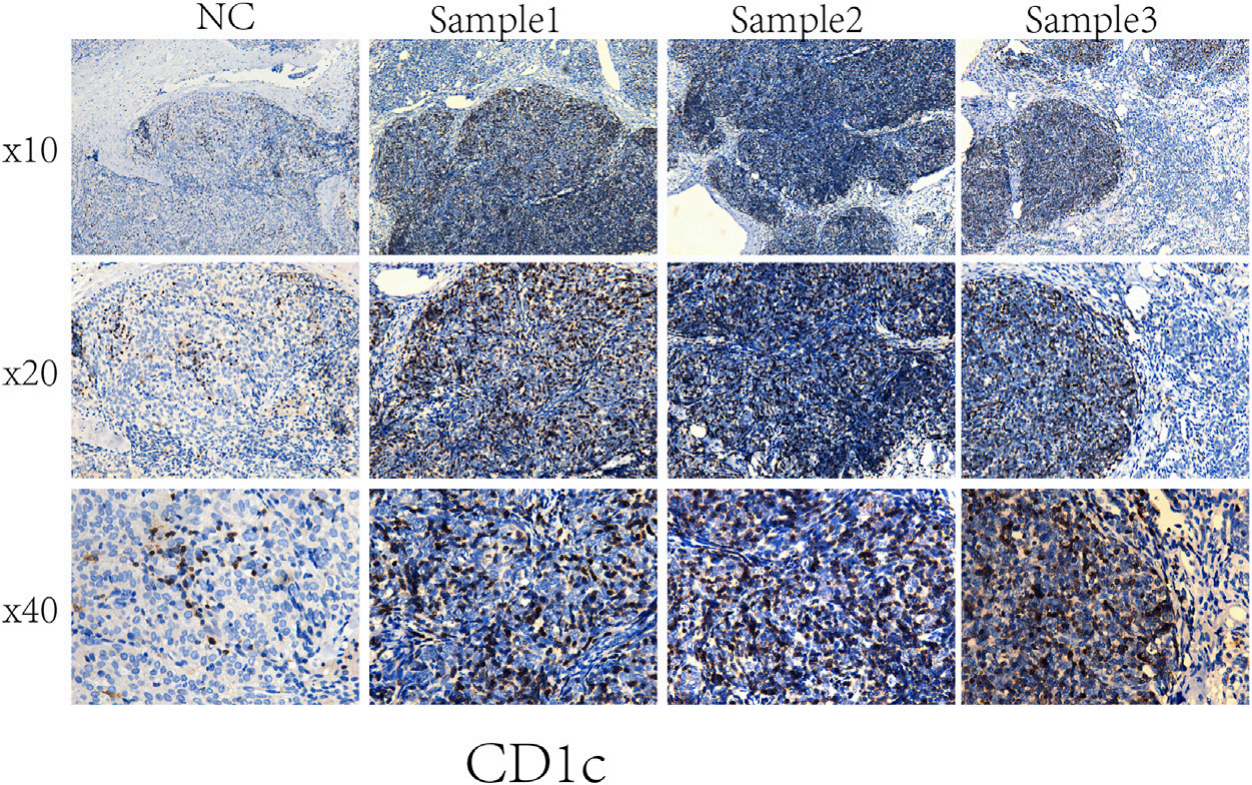
Representative results of immunohistochemistry show the expression of CD1C in THYM and normal tissues. Original magnification: ×200. **(A)**: CD1C immunostaining of THYM, **(B)**: CD1C immunostaining of normal tissues.

To compare the tumor microenvironment characteristics between the high- and low-risk score groups, the proportions of immune cells were analyzed. The results showed that various immune cells were significantly different between the high- and low-risk score groups, including activated dendritic cells, resting dendritic cells, M0 macrophages, M1 macrophages, M2 macrophages, resting mast cells, activated mast cells, resting NK cells, activated NK cells, naïve CD4 T cells, gamma and delta T cells, and regulatory T cells (Figure 8A). These different proportions of immune cells were further analyzed via correlation analysis. Some immune cells were closely related, such as activated M1 macrophages and NK cells, activated M2 macrophages and NK cells, resting mast cells and activated NK cells, and M2 macrophages and resting dendritic cells (Figure 8B), which suggests cross-talk or relevant signaling pathways between different immune cells. The risk score of the prognostic model in THYM was positively correlated with the TMB score (Figure 8C and Supplementary Table S13), indicating that the high-risk score group might have a poor prognosis accompanied by a high TMB score (r = 0.7443, *p* < .0001). The risk score of the prognostic model in THYM was also positively correlated with the ERGE-mRNAsi score (Figure 8D and Supplementary Table S14), indicating that the high-risk score group might have higher stemness ability (r = 0.2419, *p* = .0086). Some DEGs identified in the prognostic model of THYM revealed a significant correlation between their expression and sensitivity to drugs including Vinblastine, PX-316, Nelarabine, Bendamustine, Asparaginase, Cladribine, Chlorambucil, Rebimastat, Fludarabine, XK-469, Fluphenazine, Dexamethasone Decadron, and Chelerythrine. For example, there was a significant positive correlation between the expression of *TSHR* and XK-469, *TSHR* and Bendamustine, *RFX8* and Nelarabine, *CD1C* and Chelerythrine, *UGT3A2* and Chelerythrine, *TSHR* and Nelarabine, *UGT3A2* and Nelarabine, and *CD1C* and Nelarabine, and there was a significant negative correlation between the expression of *HKDC1* and Vinblastine (Figure 9 and Supplementary Table S15). These results provide potential clues for the future development of drugs for THYM treatment. Moreover, we selected CD1C as a representative to verify its expression between THYM tissues and normal tissues, and immunohistochemistry the results showed that CD1C was overexpressed in THYM tissues (Figure 10).

## Discussion

Thymic epithelial cells can undergo abnormal differentiation and gradually form the anterior mediastinum tumor-THYM. The pathogenesis of THYM is complex and unclear, although certain viral infections have been reported to be associated with the tumorigenesis of THYM (Gong et al., 2019). However, the limited clinical specimens in prior studies made it difficult to conduct in-depth basic research linking the disease phenotype and genetic background. The histological features are variable in thymic tissues, which could originate from type A to type B3, according to the proportion of lymphocytes (Sakane et al., 2019). Some patients with THYM are diagnosed at advanced stages and are ineligible for complete surgical resection. Furthermore, the chemotherapeutic regimen (cisplatin, doxorubicin, cyclophosphamide, prednisolone, doxorubicin, vincristine, etoposide, carboplatin, and paclitaxel) used in clinics sometimes yields unsatisfactory results (Hofmann and Ried, 2019). Immunotherapy has contributed to the treatment of multiple other malignancies and has proven to be a promising therapeutic alternative. Thus far, accumulating evidence on immune checkpoints and abundant PD-L1 expression has been reported in THYM in several studies, and some studies have further evaluated the clinicopathological and prognostic significance of PD-L1 (Bedekovics et al., 2020). Case reports have indicated that some patients with THYM responded well to agents targeting the PD-1/PD-L1 pathway (Arbour et al., 2017). Some phase I and II trials on immune checkpoints are currently ongoing to assess their effectiveness. The results suggested that the PD-1 inhibitor exhibits acceptable clinical efficacy for THYM; however, concerns regarding immunological adverse events cannot be ignored (Yokoyama and Miyoshi, 2018). To optimize therapeutic agents for immune checkpoints, additional studies are necessary to explore the immune microenvironment and characterize its basic mechanisms. This study revealed a reliable gene signature significantly related to prognosis and TME cell infiltration in THYM, which can promote individualized treatment and provide potential novel targets for immunotherapy.

To our knowledge, this is the first study to explore the association among clinical index, cancer cell stemness, and TMB based on the immune microenvironment in THYM. Increasing evidence showed that immune related cells, immune related molecules, immune related cytokines, and immune related pathways were closely associated with malignant biological behavior of carcinoma. The classification of tumor antigen, antitumor and application of immunotherapy, and mechanism of tumor immune escape became a research hotspot in immunotherapy strategy. The development and progression of malignant tumors is multifactorial, so the systematic analysis of immune cells, stemness, and gene mutation might help researchers to better understand the complex nature of the disease. In total, 707 DEGs were identified between immunotypes A and B of THYM, including 80 IRGs and 177 OS-related DEGs. Some of the identified DEGs have already attracted great attention in the carcinogenesis and development of THYM or have been reported as tumor biomarkers. For example, myasthenic syndrome is an important clinical symptom in patients with THYM. The mutation of the differentially expressed muscle skeletal receptor tyrosine kinase (MUSK) identified in this study is clearly associated with myasthenic syndrome. The common clinical complaints of patients with high MUSK antibodies include oculo-bulbar or respiratory crisis (Nagappa et al., 2019). A meta-analysis summarized the relationship between potential biomarkers and malignancy of THYM and suggested that mesothelin (MSLN) has a significant relationship with tumor proliferation and degree of malignancy (Zeng et al., 2020). *NPY* encodes a neuropeptide (neuropeptide Y) that is widely expressed in the nervous system and is associated with various metabolic diseases and influences many physiological processes, such as food intake, cardiovascular function, circadian rhythm, stress response, and cortical excitability. The distributive patterns of neuropeptide Y of the human thymus were observed in normal and THYM mice. *NPY*-positive fibers were widely distributed in the perivascular and parenchymal sites of several thymus lobular zones. However, the distribution of *NPY*-positive fibers (perivascular and parenchymal sites) was quantitatively reduced in THYM when compared with several thymus lobular zones (Mignini et al., 2011). Some of the differentially expressed IRGs play roles in tumor immunity in THYM. For example, several drug candidates have been reported to exert strong inhibitory effects on *IL-4* expression. When the *IL-4* gene promoter is inhibited in EL4 T THYM cells, the generation of specific immunoglobulin E (IgE) is influenced in clinical settings (Park et al., 2006). The differentially expressed IRG *IL1* was reported to induce rapid and sustained activation of Rac1 in the THYM cell line EL4 (Jefferies and O’Neill, 2000). IL-8 has been developed as a new diagnostic biomarker for the identification and recurrence surveillance of THYM. IL-8 levels were markedly increased in patients with THYM in naïve T cells, and significant clinical relevance with clinicopathological features was observed (Gao et al., 2020). These examples of identified DEGs and IRGs in THYM indicate that some findings are consistent with those of previous studies. Simultaneously, some new findings were also obtained that provide a direction for future studies.

The 707 identified DEGs in this study were significantly enriched in some crucial immune and cancer-related pathways. This study focused on some traditional pathways. For example, previous research has suggested that THYM might be related to type I T-cell virus infections or the Epstein–Barr virus (Erkmen et al., 2011). Viral carcinogenesis has been identified as an abnormal pathway, and the enriched DEGs include *HIST1H2BM, HIST1H2BO, HIST1H4K, ACTN3, HIST1H4L, HIST1H2BI, HIST1H2BL, HIST1H4A, HIST1H4B, CREB3L3, HIST1H2BH, HIST1H2BB, HIST1H4C, HIST1H4D,* and *HIST1H4F.* The enriched genes are key components of histones; among them, *HIST1H2BH* was reported to participate in transcriptional mis-regulation in cancer pathways by regulating the response to catecholamine stimulus, limbic system development, and catecholamine metabolic process (Tian et al., 2019). The histone-related gene *HIST1H4F* was hypermethylated in various cancers from TCGA datasets (*n* = 7344). Moreover, some types of cancer have been verified to include hypermethylated *HIST1H4F*, which demonstrates that *HIST1H4F* can be developed as a universal cancer-only methylation marker (Dong et al., 2019). The discovery of crucial biomarkers in related pathways may aid in understanding general tumorigenesis and the intrinsic mechanism. Transcriptional mis-regulation in the cancer pathway was another crucial pathway identified in the present study. Proteins of the matrix metalloproteinase (MMP) influence the physiological processes of reproduction, tumor metastasis, and embryonic development via breakdown of the extracellular matrix (Li et al., 2020). The mRNA expression of the *MMP* gene was obtained from lymphocytes, epithelial tumor cells, and stromal cells of invasive THYM using laser-capture microdissection. The results showed that mRNA expression levels were significantly correlated with the clinical stage (stage I–II vs. stage III–IV) and histological subtype of THYM (Wang et al., 2007). Granulocyte-macrophage colony-stimulating factor (CSF) controls the production, differentiation, and function of granulocytes and macrophages. One research group studied the function of CSF in non-lymphohematopoietic malignant tumors accompanied by leukocytosis, and CSF-positive cases were found in malignant THYM. CSF-positive events were significantly associated with malignant tumors accompanied by leukocytosis (Kojima et al., 2002). The next crucial pathway was cytokine-cytokine receptor interaction, and the enriched genes included *TNFSF18, CSF2, TNFSF15, CCL20, INHBA, PRL, IL4, IL1A, CCL7, THPO, IFNE, CCR9, IL12A, PF4V1,* and *CCL15.* The present study found that micronodular THYM contained a high proportion of monoclonal B-cell populations. Simultaneously, the neoplastic epithelium of micronodular THYM expressed high levels of dendritic cells and T-cells. Furthermore, the results suggest that abnormal chemokine expression of CCL20 might take part in promoting the recruitment of thymic mucosa–associated lymphoid tissue lymphomas and inducing B-cell homeostasis in micronodular THYM (Ströbel et al., 2005). The enriched pathways identified in the present study might help to elucidate the pathogenesis of THYM, and some crucial DEGs enriched in these pathways could be potential biomarkers or drug targets.

Owing to recent developments in molecular biology, the relatively well-developed TCGA database provides gene data (gene mutation, transcriptome analysis, and epigenetic analysis) and the corresponding clinical data (Li et al., 2019). An increasing number of data-mining techniques have been applied to investigate novel biomarkers and potential mechanisms in tumorigenesis and tumor development. In this study, DEGs were identified in THYM based on differences in immune status, and an 11-gene signature prognostic model and risk score were constructed via lasso regression analysis. The prognostic model (*CELF5, ODZ1, CD1C, DRP2, PTCRA, TSHR, HKDC1, KCTD19, RFX8, UGT3A2,* and *PRKCG*) was verified by PCA, ROC, and GEO data. The indicated risk scores could be a novel pattern for predicting the OS rate of patients with THYM. Because the pathogenesis of THYM is complex, a single-gene or traditional model is not appropriate. The multiple-gene signature represents a new theoretical paradigm in the study of tumors (Li and Zhan, 2020). The selected genes in the prognostic model are consistent with previous reports on their functions in immunity and cancers. For example, the transmembrane glycoprotein family, CD1C, is structurally related to the major histocompatibility complex proteins. The CD1C proteins participate in mediating the presentation of glycolipid antigens and primary lipids of T cells throughout the endocytic system (Visvabharathy et al., 2020). As one of the important identified genes in the prognostic model, CD1C was overexpressed in THYM tissues showed by immunohistochemistry. It is the first time to identify the high expression of CD1C in THYM, and to some extent our result was consistent with the previous study in other diseases. For example, CD1C+ immune cells were used for immunotherapy of metastatic hormone refractory prostate cancer (PMID: 25658616). Expression of CD1C enhances human invariant NKT cell activation by α-GalCer, which indicated that B cell neoplasias that co-express CD1C and CD1D may be particularly susceptible to α-GSL therapy, and cancer vaccines using α-GSLs as adjuvants may be most effective when presented by CD1C+ antigen-presenting cells (PMID: 23885215). *CELF5* can regulate pre-mRNA alternative splicing, mRNA editing, and translation, helping to generate more alternatively spliced variants and induce diversity of antigens (Huang et al., 2019). DRP2, a member of the dystrophin family, which resembles certain short C-terminal isoforms of dystrophin protein, plays a critical role in the maintenance of membrane-associated complexes. DRP2 is a crucial molecule in extracellular matrix-receptor interaction pathways and has been reported to be related to cancer development (Bao et al., 2019). Additionally, the association among immune cells, TMB, stemness, and drug sensitivity was also an important part of the present study. Understanding the differences in the immune characteristics of THYM between high- and low-risk score groups would help to optimize immunotherapy strategies in a number of human clinical trials. Investigating the significance of immune cells was meaningful for the further study of tumor immunity. For example, patients with THYM had a significantly higher percentage of regulatory T cells and a lower percentage of CD4^+^ naïve T cells compared with healthy controls. Aberrant immunologic disorders and altered T-cell subsets in THYM were significantly associated with clinical phenotype and prognosis (Thongprayoon et al., 2013). TMB appears to be a new biomarker for predicting the effects of and immune response to immunotherapy. For example, in patients with lung cancer treated with anti-PD-1/PD-L1 immune checkpoint inhibitors, TMB was positively correlated with the objective response rate, progression-free survival, and clinical benefit time (Sha et al., 2020). TMB is still being explored as a biomarker in immunotherapy. Stemness in tumors induces infinite proliferation, self-renewal, and relapse and is a great challenge for tumor therapy (Denton et al., 2018). In this study, the calculated risk score was positively correlated with stemness index, indicating that some subtypes of THYM might comprise sorted cancer stem cells. Importantly, the correlation analysis of DEG expression in the prognostic model showed that some genes were significantly associated with drug sensitivity. Some drugs have been reported to be used in patients with THYM; for example, a patient who was treated with combined chemotherapy (vinblastine) has been doing well and did not show recurrence after operation (Shundo et al., 2004). These results provide potential directions for the future development of drugs.

## Conclusion

We systematically plotted the distribution of immune cells and immune related genes in THYM and the relationship with clinicopathological characteristics. The different immunophenotypes showed differences in gene expression, distinct signaling pathways, and special biological processes. THYM, which affects an important immune organ, is associated with the abnormal expression of PD-L1, which makes the exploration of the tumor immune microenvironment meaningful. A gene signature for the prognostic model represents a potentially useful tool in THYM; however, verification in clinical practice with a large number of patients is needed. Nevertheless, the present findings may help to elucidate the pathogenesis and immunophenotype of THYM in the future.

## Data Availability

The datasets presented in this study can be found in online repositories. The names of the repository/repositories and accession number(s) can be found in the article/Supplementary Material.

## Abbreviations

THYM, thymoma; DEGs, differentially expressed genes; IRG, immune-related genes; KEGG, kyoto encyclopedia of genes and genomes; GO, gene ontology; PPI, protein-protein interaction; TMB, tumor mutation burden; OS, overall survival.
